# Gallstone Ileus as an Infrequent Cause of Bowel Obstruction: A Review of Small Cohort

**DOI:** 10.7759/cureus.58438

**Published:** 2024-04-17

**Authors:** Shashi Kumar, Qasif Qavi, Nida Bashir, Firas Alkistawi, Omotara Lesi, Praveen Sekaran, Jafer Hamdani, Abdalla Saad Abdalla Al-Zawi

**Affiliations:** 1 Surgery, Mid and South Essex NHS Foundation Trust, Basildon, GBR; 2 Surgery, Basildon and Thurrock University Hospital, Basildon, GBR; 3 General Surgery, Mid and South Essex NHS Foundation Trust, Basildon, GBR; 4 General and Colorectal Surgery, Basildon University Hospital, Basildon, GBR; 5 General Surgery, Basildon and Thurrock University Hospital, Basildon, GBR; 6 General and Breast Surgery, Mid and South Essex NHS Foundation Trust, Basildon, GBR; 7 General and Breast Surgery, Anglia Ruskin University, Chelmsford, GBR

**Keywords:** cholecystoenteric fistula, rigler's triad, karewsky's syndrome, exploratory laparotomy, enterolithotomy, intestinal obstruction, gallstone ileus, gallstones

## Abstract

Introduction

Gallstone ileus is an uncommon cause of small bowel obstruction; it is a rare complication of calculus chronic cholecystitis which leads to cholecystoenteric fistula and impaction of gallstone in the gastrointestinal tract leading to mechanical bowel obstruction. Our aim is to report the natural history and management of this rare condition in a teaching hospital.

Materials and methods

It is a retrospective study, where 10 years of data related to the management of intestinal obstruction secondary to gallstone ileus was collected. The cohort included 10 patients, whose demographic data, clinical findings, and management outcomes were evaluated.

Results

Majority of patients were female (90%, n=9) with a median of 83 years (range 61-96) although 90% of the population were above 70 years. Presenting complaints were mostly pain and vomiting. The onset of symptoms was between two and seven days. The site of obstruction was mostly the ileum (n=9) with the exception of one case in the sigmoid proximal to a benign stricture, and the size of the stone ranged from 2.5 to 4 cm. Moreover, most of the patients had a previous history of gallstone (n=7) with one post-cholecystectomy status. The laboratory investigations in 50% of patients had deranged liver function test (LFT) and acute kidney injury (AKI), and 60% had raised inflammatory markers, namely, white blood cells (WBC) and C-reactive protein (CRP). Intervention as enterolithotomy was the preferred approach (n=8 (two laparoscopic, six open surgery)), and two patients were managed conservatively. The mean postoperative length of stay was 10 days in the open approach and five days in the laparoscopic approach, respectively.

Conclusions

Elderly female patients are more prone to have gallstone ileus particularly with a past medical history of gallstones, and the preferred management option is enterolithotomy which could be open or laparoscopic depending on the expertise of the surgeon.

## Introduction

Gallstone ileus is an infrequent cause of intraluminal mechanical bowel obstruction. It is regarded as one of the uncommon complications of cholelithiasis causing 1-4% of bowel obstruction in the adult population [1-4]. It was first described by Thomas Bartholin in 1654 in an autopsy. The first cause of duodenal obstruction due to gallstones was described preoperatively by French surgeon M. Beassier in 1770. However, subsequently, L. Bouveret, a French physician, published two comprehensive case reports in 1896 on this condition, and it was named after him [1,5]. The name gallstone ileus is a misnomer because, by definition, ileus means non-mechanical bowel motility failure, although this condition is a mechanical obstruction of the bowel by a stone. Gallstone ileus occurs when a gallstone passes into the gastrointestinal tract (GIT) through the bilioenteric fistula as a result of recurrent acute cholecystitis with widespread chronic inflammation and adhesions between the gallbladder and digestive tract (duodenum, most common, stomach, jejunum, and colon) which leads to ischemia/necrosis of the intestinal wall and eventually fistula formation with migration of the stone from the gallbladder to the GIT [1,3,5]. Gallstone ileus has three forms of clinical presentation: acute, the classical gallstone ileus; subacute, as partial bowel obstruction; and chronic, known as Karewsky's syndrome, which is characterized by recurrent episodes of abdominal pain due to the passage of gallstones lodged in the bowel lumen with interspersed asymptomatic long lapses [5,6]. Computed tomography (CT) scan is the imaging modality of choice with an overall 93% sensitivity, 100% specificity, and 99% diagnostic accuracy. CT sometimes reveals bilio-digestive fistula as well. CT also clearly picks up Rigler's triad (pneumobilia, intestinal obstruction, ectopic gallstone) in most cases [1,5-7]. Its differential diagnosis may include adhesions, ileus, ingested foreign body, as well as the not uncommon bowel malignant tumors [8,9]. Treatment of gallstone ileus can be either enterolithotomy with or without cholecystectomy and fistula closure as a one-stage procedure [2,4,10]. The aim of this study is to analyze the natural history and management of gallstone ileus in our hospital.

## Materials and methods

It is a retrospective study including a small cohort of patients diagnosed with gallstone ileus at Basildon University Hospital MSE Trust Essex-UK over 10 years from 2011 to 2021; 10 cases were identified and included. The data was collected from the patients' electronic record database. This included patients' demographics, clinical presentation, laboratory investigations (inflammatory markers, liver function tests (LFTs), and renal function tests), and radiological findings as well as the management provided. Also, the authors have paid attention to the symptoms duration, stone impact sites, stone size, clinical comorbidities, intraoperative findings, length of postoperative hospital stay, as well as the associated postoperative morbidity which was categorized according to the Clavien-Dindo classification. The quantitative variables (as laboratory findings) were defined through medians and percentiles and the qualitative variables (such as age, gender, or medical history) by frequency and percentage. Clinical data collection and statistical analysis were performed following the institutional guidelines and ethical standards of the Helsinki Declaration.

## Results

We retrieved clinical data of 10 patients during this time period; no patients were excluded. Among these, nine were females, and one was a male. The median age of diagnosis was 83 years (range: 61-96 years); seven patients (70%) had a history of previous gallstone disease. The median American Society of Anesthesiologists (ASA) score was 3 (range: 3-4). Associated comorbidities are reported in Table 1.

**Table 1:** Demographic data and comorbidities ASA: American Society of Anesthesiologists; HTN: hypertension; DM: diabetes mellitus; IHD: ischemic heart disease; AF: atrial fibrillation

	Overall (n=10)	Operated (n=8)	Not operated (n=2)
Mean age (year)	81.6 (61-96)	80.8 (61-96)	84.5 (75-94)
Female/male	9/1	7/1	2/0
Mean ASA	3	3	4
Comorbidity N(%)			
HTN	10	8	2
DM	2	1	1
Stroke	4	2	2
Cardiopathy (IHD, AF)	4	2	2
Ulcerative colitis	1	0	1
Lung disease	1	0	1
History of previous gallstone	7	5	2
Previous abdominal surgery (N)	3	2	1
Sleeve gastrectomy	1	1	0
Laparoscopic cholecystectomy	1	1	0
Subtotal colectomy	1	0	0

Clinical presentation was typical of intestinal obstruction with abdominal pain in all cases, vomiting in eight cases, and bowels not opened (BNO) in four cases. The time of onset of symptoms until presentation to the emergency department ranged from two to seven days with a median of three days (Table 2).

**Table 2:** Clinical presentation

	Overall (n=10)	Operated (n=8)	Not operated (n=2)
Abdominal pain	10 (100%)	8 (100%)	2 (100%)
Vomiting	8 (80%)	8 (100%)	0 (0%)
Constipation	4 (40%)	2 (25%)	2 (100%)
Duration of onset (median)	3 (2-7)	35 (2-7)	2.5 (2-3)

Laboratory investigation showed that 60% of the patients had raised C-reactive protein (CRP) of a mean of 104.16 mg/L (range: from 17 to 215 mg/L). A leukocytosis of a mean of 15.28×109/L (range: from 14.4 to 15.8×109/L) was detected in 50% of cases. Also, 50% of the patients reported having acute kidney injury (AKI) and deranged LFTs as mentioned in Table 3.

**Table 3:** Laboratory investigations WBC: white blood cell; CRP: C-reactive protein; LFT: liver function test; ALP: alkaline phosphatase

	Overall (n=10)		Operated (n=8)	Not operated (n=2)
↑WBC				
N(%)		5 (50%)	4 (50%)	1 (50%)
Mean (range)×10^9^/l		15.28 (14.4-15.8)	15.15 (14.4-15.8)	15.8
↑CRP				
N(%)		6 (60%)	5 (62.5%)	1 (50%)
Mean (range) mg/l		104.16 (17-215)	83 (17-215)	210
↑LFT				
N(%)		5 (50%)	4 (50%)	1 (50%)
↑bilirubin		3 (30%)	2 (25%)	1 (50%)
Mean (range) umol/l		35 (30-45)	37.5 (30-45)	30
↑ALP		3 (30%)	2 (25%)	1 (50%)
Mean (range) IU/l		153.33 (134-169)	145.5 (134-157)	169
↑creatinine				
N(%)		5 (50%)	5 (62.5%)	0 (0%)
Mean (range) umol/l		143 (115-189)	143 (115-189)	0 (0)

CT scan was performed in all cases for preoperative diagnosis with findings summarized in Table 4.

**Table 4:** CT scan radiological findings CT: computed tomography

	Overall (n=10)	Operated (n=8)	Not operated (n=2)
Rigler's triad	9 (90%)	7 (87.5%)	2 (100%)
Site of impacted stone			
Ileum	9 (90%)	8 (100%)	1 (50%)
Sigmoid	1 (10%)	0 (0%)	1 (50%)
Bilioenteric fistula			
Cholecystoduodenal	5 (50%)	4 (50%)	1 (50%)
Cholecystocolonic	1 (10%)	0 (0%)	1 (50%)

**Figure 1:**
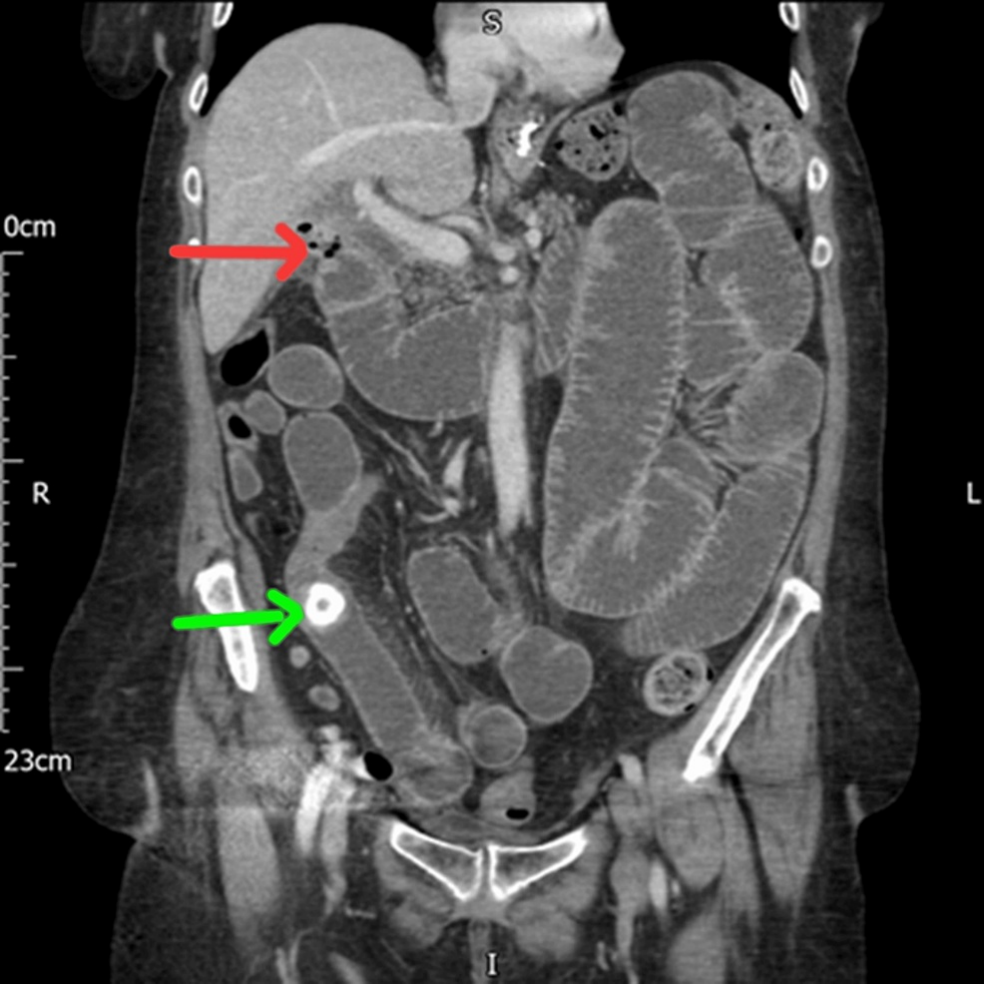
CT of the abdomen coronal view showing stone in the terminal ileum (the green arrow) and cholecystoduodenal fistula (the red arrow) CT: computed tomography

Rigler's triad was found in 90% of the patients and bilio-digestive fistula in 60%, which includes cholecystoduodenal (50%) (Figure 1).

**Figure 2:**
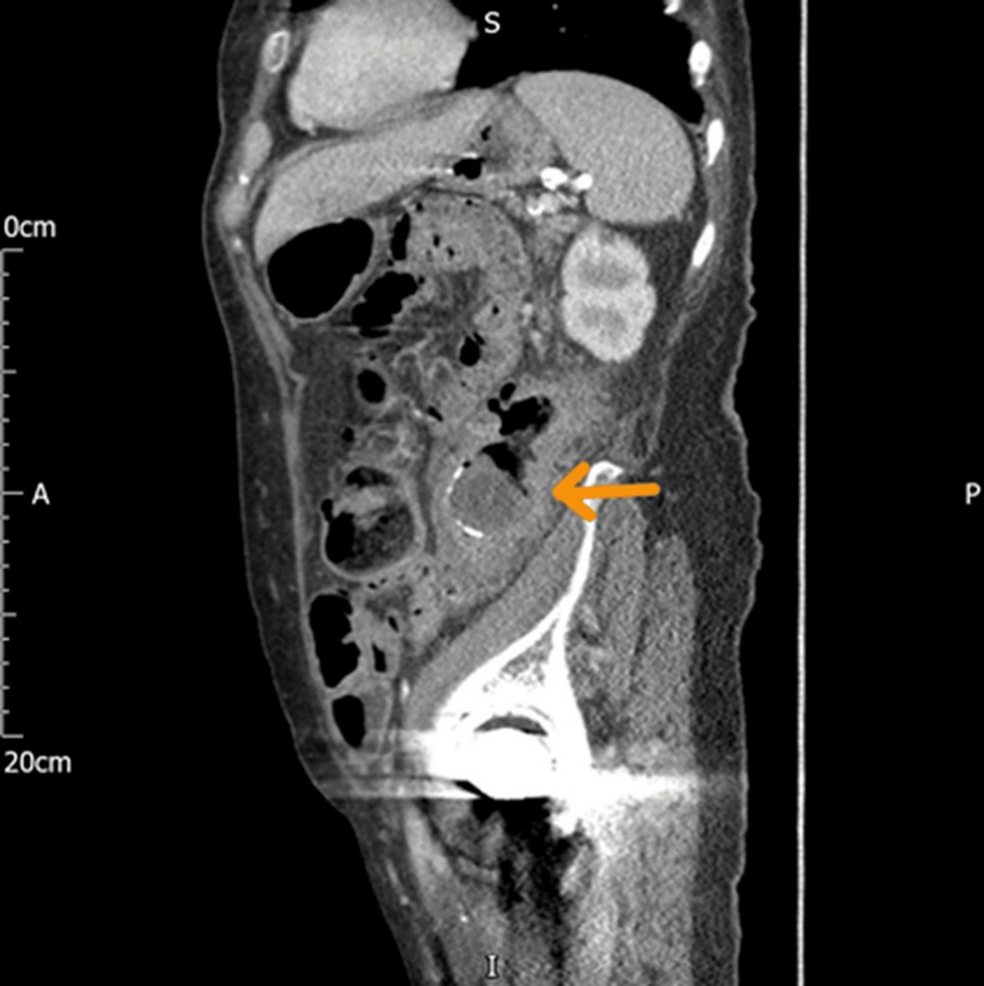
CT of the abdomen sagittal view shows gallstone in the colon (the brown arrow) CT: computed tomography

Also, the imaging reported the site of obstruction with impacted stone in the ileum in 90% (Figure 1) and located in the sigmoid colon (Figure 2).

**Figure 3:**
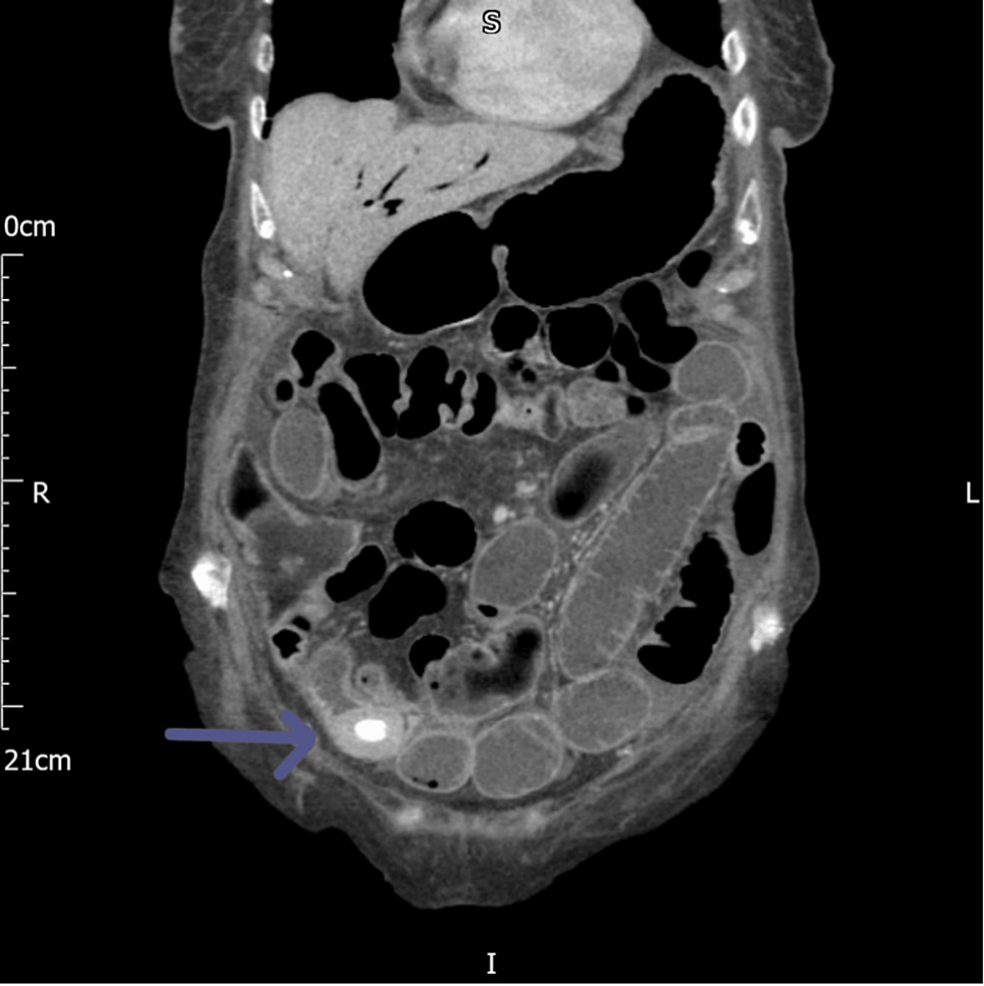
CT of the abdomen coronal view showing gallstone in the terminal ileum (the blue arrow) CT: computed tomography

**Figure 4:**
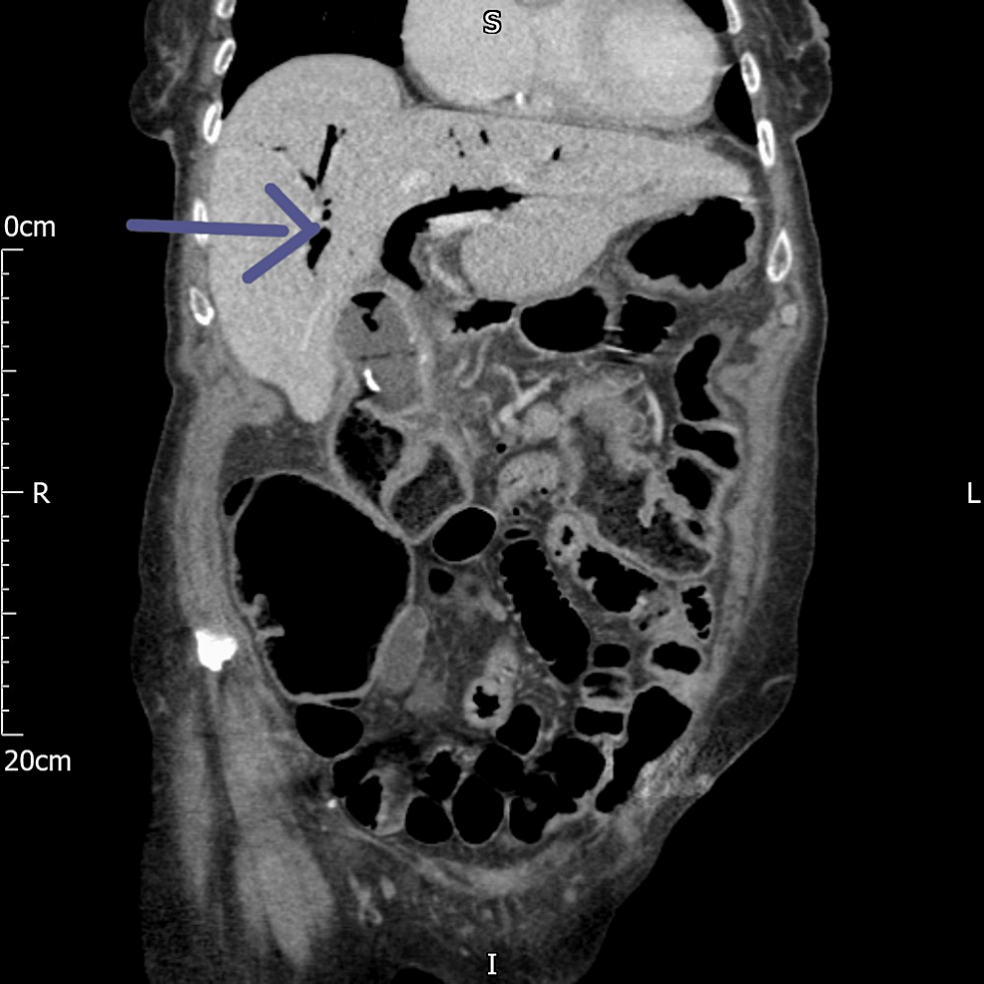
CT of the abdomen coronal view showing extensive pneumobilia (the blue arrow) CT: computed tomography

The pneumobilia was seen in 90% of cases (Figure 3 and Figure 4).

In our study, eight patients were treated surgically, where six patients underwent laparotomy and enterolithotomy, while the other two had laparoscopic enterolithotomy. Out of eight operated patients, six patients had findings of a single stone, and the other two patients had double stones retrieved from the small bowel. The size of stones ranged from 2.5 cm to 4 cm with a mean of 3.25 cm. Intraoperatively, six patients had stones impacted in the terminal ileum, and two had mid-ileum impactions with proximal bowel dilatation (Table 5).

**Table 5:** Intraoperative findings in gallstone ileus cases

	Overall (n=8)	Open surgery (n=6)	Laparoscopy (n=2)
No. of impacted stones			
One	6	5	1
Two	2	1	1
Site of bowel obstruction			
Terminal ileum	6	5	1
Mid-ileum	2	1	1
Stone size/cm (mean, range)	3.25 (2.5-4)	3 (2.5-4)	4 (4-4)

Of the non-surgically treated cohort, one was managed conservatively, and one was palliated who died eventually. Two patients of the operated group had a history of previous abdominal surgery (sleeve gastrectomy five years back and interestingly laparoscopic cholecystectomy 15 years earlier), and one patient who was managed nonoperatively had a subtotal colectomy and ileostomy as past surgical history. The overall morbidity due to post-op complications is 80%, which was assessed as per the Clavien-Dindo classification as in Table 6.

**Table 6:** Postoperative events SSI: surgical site infection; AKI: acute kidney injury; CCF: congestive cardiac failure

Event (Clavien-Dindo classification)	Clavien-Dindo classification	Overall (n=8)	Open surgery (n=6)	Laparoscopy (n=2)
SSI	II	2	1	1
Chest infection	II	2	2	0
Ileus	II	1	2	0
AKI	II	4	4	0
CCF	IVa	1	1	0

One patient had grade IVa for which the patient was managed in an intensive care unit and had a prolonged stay in the hospital. The length of hospital stay range was 3-25 days with a mean stay of 10 days in open and five days in laparoscopic surgery. However, patients who were managed conservatively were discharged three days after the resolution of symptoms, and palliative patients died after 11 days of treatment. There was no 30-day mortality reported in the operative group of patients.

In a follow-up period of 36 months, no recurrence of gallstone ileus was reported. One patient who had open enterolithotomy presented with adhesional small bowel obstruction two years after surgery for which he underwent a laparotomy and adhesiolysis. The patient who was managed conservatively presented with pain in the abdomen and deranged LFT one year after discharge, for which he had a magnetic resonance cholangiopancreatography (MRCP) and subsequently endoscopic retrograde cholangiopancreatography (ERCP) for the retrieval of a common bile duct stone.

## Discussion

Gallstone is an uncommon cause of intestinal obstruction. Most of the patients usually have a history of gallstone disease with chronic cholecystitis and chronic inflammation, which creates communication with the adjacent bowel loop leading to bilioenteric fistula and migration of stone from the gallbladder to the lumen of the bowel. These stones gradually increase in size with the addition of intestinal content until they reach the point of impaction, and this chance is more likely if the stone is larger in size. In our study, the average size of the stone was more than 3 cm. It is predominantly a disease of the female population in their late age. In our study, nine patients were above the age of 70 years, and 50% were above 80. Clinical presentation is not specific. All patients essentially present with signs and symptoms of intestinal obstruction. Patients present with pain in the abdomen, vomiting, abdominal distension, or constipation depending on the site of impaction of the stone which can be small bowel or large bowel. Pain in the abdomen is attributed to both obstruction and recent inflammation of the gallbladder. In our study, abdominal pain was encountered in all cases. For intestinal obstruction, the sites affected usually have narrowing in the bowel lumen; the most common are the terminal ileum and ileocaecal valve (60-75%), jejunum and proximal ileum (20-40%), sigmoid colon (4%), and duodenum (3-10%) [2,3,6,8,11,12]. In our study, 70% had a stone in the terminal ileum, 20% in the proximal ileum, and only 10% in the sigmoid colon. Rare symptoms may result from the impaction of stone at sites of strictures, such as Crohn's disease or diverticulitis, or at the neck of Meckel's diverticulum as has been described [12,13]. Abdominal distention presents in 84% of cases [8], and Bouveret's syndrome is a recognized presentation, where the gallstone causes gastric outlet obstruction due to stone impaction in the duodenal bulb and patients present with nausea, vomiting, abdominal pain, and hematemesis [14,15]. Laboratory blood investigation does not follow any specific trend apart from raised inflammatory markers, infrequent mild deranged LFTs, and raised creatinine as revealed in two Mexican case series studies and other reports [2,5,6]. CT scan is the best modality of investigation for preoperative diagnosis [16]. The presence of pneumobilia/aerobilia, ectopic stone in the intestine, and intestinal obstruction was seen in the majority of the patients, referred to as Rigler's triad, named after the radiologist who first described these findings on plain film in 1941 [2,5,8,17,18]. Yu et al. in 2005 had reviewed 165 CT images which belonged to 14 cases of gallstone ileus. They have mentioned that gallstone ileus CT scan diagnostic criteria are (1) bowel obstruction; (2) ectopic gallstone, either rim calcified or total calcified; and (3) abnormal gallbladder with complete air collection, presence of air-fluid level, or fluid accumulation with an irregular wall [19]. In our study, classical bilioenteric fistula was seen in more than 50% of the cohort as compared to the demonstration of bilioenteric fistula in 80% of cases in the Kasahara et al. study [20]. The differential diagnosis of gallstone ileus includes adhesional bowel obstruction in patients with a previous history of abdominal surgery/radiotherapy and ingested foreign body. Remembering that bowel malignancy is occupying the third place in the newly diagnosed cancers every year as reported in the GLOBOCAN (World Health Organization Global Cancer Institute) database, it is another differential diagnosis that has to be considered especially in the older age group presenting with acute abdominal symptoms [21]. The chance of spontaneous resolution of symptoms of gallstone ileus is unlikely. Surgical management remains the standard approach to deal with intestinal obstruction, although there is no standard definitive surgical technique [1,2,3,5,10,22]. This disease is usually seen in the elderly population who are more likely to have multiple comorbidities; however, surgery is not always feasible for these groups of patients [2]. The aim of an operation would be immediate relief of obstruction which is enterolithotomy. It is mostly dealt with by open emergency laparotomy operations; however, laparoscopy is also an acceptable treatment option in expert hands as it reduces morbidity and postoperative complications [12,23]. Few studies have advocated one-stage treatment of enterolithotomy with cholecystectomy and fistula repair, but the author believes that one-stage surgery may not possibly be in the best interest of some patients as surgery in itself carries an inherent risk of bleeding and infection. In addition, cholecystectomy may result in inadvertent injury to the biliary tree, especially in the presence of chronic inflammation and also when the patient is elderly with other comorbidities [1,3,5,17,23]. Some authors recommend attempting endoscopic removal of the stones for stones located in the stomach and duodenum; aside from this, a colonoscopy can be performed for the diagnosis and even treatment of impacted stones in the colon and terminal ileum [24]. Alternative treatment options are also available in gallstone ileus which include extracorporeal shock wave lithotripsy (ESWL) and endoscopic hydroelectric laser lithotripsy, although these methods are only possible in stones lodged in the colon, proximal duodenum, or stomach [8,15]. The author advocates that all postoperative patients after gallstone ileus should be educated before discharge from the hospital on potential complications of bilioenteric fistula which include recurrence of bowel obstruction, biliary sepsis, and acute pancreatitis. They should be informed to attend the emergency department in case of severe abdominal pain and vomiting. The main limitation of our study was its retrospective design and its small sample size; however, the low incidence of gallstone ileus in the general population is well known. This condition is reported in 0.3-0.5% of patients with gallstones [12,16] and among 1-4% of patients with bowel obstruction [1,3,4]. The small cohort may not yield an accurate estimate, in particular p-values or confidence intervals.

## Conclusions

Gallstone ileus is primarily a disease of advanced-aged women with associated comorbidity. We should suspect this disease in the older population who present with intestinal obstruction, especially those who are known to have gallstone disease. CT scan is the best modality to establish the preoperative diagnosis. There are no randomized controlled trials (RCT) or level 1 evidence to support optimal therapy, but we recommend enterolithotomy as a first-line safe approach to manage this condition. Surgical management could be open or laparoscopic depending on the expertise of the operating surgeon although laparoscopy clearly reduces associated morbidity and postoperative stay in the hospital.
